# A 12 month randomised comparative efficacy trial into the treatment of calcaneal apophysitis

**DOI:** 10.1186/1757-1146-8-S2-O22

**Published:** 2015-09-22

**Authors:** James Alicia, Williams Cylie, Haines Terry

**Affiliations:** 1 Podiatry Department, Peninsula Health Service, Frankston, Vic, 3199, Australia; 2 Physiotherapy Department, Monash University, Frankston, Vic, 3199, Australia; 3 Allied Health Research Unit, Monash Health, Cheltenham, Vic, 3192, Australia

## Background

There are many clinically provided treatment for calcaneal apophysitis however there is very little evidence supportive the comparative efficacy between these. This study compared the efficacy of currently employed treatment options for the relief of pain and disability associated with calcaneal apophysitis.

## Method

This is the world's largest randomised comparative efficacy trial with one, two, six and 12-month follow-up. Children with clinically diagnosed calcaneal apophysitis (n = 124) were recruited. There were two treatment factors within the study. Factor 1 was two different types of in-shoe orthoses: a heel raise or prefabricated orthoses. Treatment factor 2 was footwear replacement. The alternate condition in this factor was no footwear replacement. These interventions were also representative for different hypothesized causative mechanism of calcaneal apophysitis.

The primary outcome was pain/disability measured using the Oxford ankle foot questionnaire. The secondary outcomes were pain measured using the Faces pain scale and ankle range of motion measured using the weight-bearing lunge test. At the six-month and 12 month follow up point the Oxford ankle foot questionnaire was completed as the single outcome measure. The intervention factors were examined over time on the primary and secondary outcome measures using a generalized estimating equation.

## Results

The children within this study experienced pain for a mean 10.87 (0.61) months prior to intervention. There was no main effect of shoe insert (heel raise v's prefabricated orthoses), or footwear (usual footwear v's athletic footwear), nor an insert by footwear interaction effect for three of the four domains of the primary outcome measure. There was a difference in the footwear domain between footwear intervention groups (usual footwear vs. athletic footwear) however the direction of this difference was opposite for children (p=0.02, favouring usual footwear) than for their parent (p=0.017, favouring athletic footwear).

## Conclusion

This study indicates that there is no relative advantage to any one of the investigated treatment choices over another. The only difference detected related to whether children felt they could wear the footwear of their choice. Therefore the selection of treatment choice may defer to a decision of cost minimization, patients preference and clinicians judgment.

